# Efficacy of erythropoietin alone in treatment of neonates with hypoxic-ischemic encephalopathy: A protocol for systematic review and meta-analysis

**DOI:** 10.1097/MD.0000000000026365

**Published:** 2021-06-18

**Authors:** Guang Yang, Zhimin Xue, Yuan Zhao

**Affiliations:** a Department of Pediatrics, Shanxi Medical University; b Neonatal Medicine, Shanxi Children's Hospital, Shanxi, China.

**Keywords:** erythropoietin, hypoxic-ischemic encephalopathy, meta-analysis, protocol, random, systematic review

## Abstract

**Background:**

Multiple clinical trials have demonstrated the safety and efficacy of erythropoietin in improving neurodevelopmental outcomes in infants with hypoxic-ischemic encephalopathy (HIE). It is undoubtedly urgent to include only randomized controlled trials (RCTs) for more standardized systematic reviews and meta-analyses. The purpose of this study is to examine whether erythropoietin reduces the risk of death and improve neurodevelopmental disorders in infants with HIE.

**Methods:**

The electronic databases of Cochrane Library, EMBASE, PubMed, and Web of Science were searched from the inception to June 2021 using the following key terms: “erythropoietin,” “hypoxic-ischemic encephalopathy,” and “prospective,” for all relevant RCTs. Only English publications were included. The primary outcome was mortality rate. Secondary outcomes included neurodevelopmental disorders, brain injury, and cognitive impairment. The Cochrane risk of bias tool was independently used to evaluate the risk of bias of included RCTs by 2 reviewers.

**Results:**

We hypothesized that group with erythropoietin would provide better therapeutic benefits compared with control group.

**OSF registration number:**

10.17605/OSF.IO/FERUS.

## 1 Introduction

Perinatal hypoxic-ischemic encephalopathy (HIE) is an important cause of neonatal encephalopathy, occurring in 1 to 3 of every 1000 births in the United States and affecting as many as 12,000 infants annually in the United States.^[1]^ About a quarter of HIE survivors show permanent neurological sequelae, such as cerebral palsy, mental retardation, blindness, and deafness. Treatment for the disease remains limited. Hypothermia, which begins within 6 hours of birth, improves prognosis. Still, 46% of babies are at risk of death or disability. New neuroprotective therapies are needed to further reduce the high risk of adverse outcomes after HIE.^[2,3]^

Erythropoietin has neuroprotective and neuroregenerative effects, which are net effects of anti-excitatory, anti-inflammatory, anti-oxidant, and anti-apoptotic effects on neurons and oligodendrocytes.^[4]^ In addition, erythropoietin promotes neurogenesis, oligodendrogenesis, and angiogenesis, which are essential for normal neurodevelopment and injury repair.^[5,6]^ These beneficial effects of erythropoietin have been well documented in experimental models of neonatal brain injury, and emerging clinical data also show benefits. The safety of high doses of erythropoietin in newborns is also reassuring.^[7]^

Multiple clinical trials have demonstrated the safety and efficacy of erythropoietin in improving neurodevelopmental outcomes in infants with HIE.^[8–10]^ A recent review involving randomized and non-randomized trials assessed neurodevelopmental disorders in infants with HIE. The authors did not explicitly define neurodevelopmental disorders, nor did they assess the risk and level of evidence for bias.^[11]^ Therefore, it is undoubtedly urgent to include only randomized controlled trials (RCTs) for more standardized systematic reviews and meta-analyses. The purpose of this study is to examine whether erythropoietin reduces the risk of death and improve neurodevelopmental disorders in infants with HIE.

## 2 Materials and methods

### 2.1 Selection of studies

The systematic review protocol was registered on Open Science Framework registries. Two independent investigators followed the Preferred Reporting Items for Systematic Reviews and Meta-Analyses reporting guidelines to conduct this study. The electronic databases of Cochrane Library, EMBASE, PubMed, and Web of Science were searched from the inception to June 2021 using the following key terms: “erythropoietin,” “hypoxic-ischemic encephalopathy,” and “prospective,” for all relevant RCTs. Furthermore, the reference lists from published original articles and relevant reviews were assessed to identify more relevant studies. Only English publications were included. Ethical approval was not necessary because the present meta-analysis was performed on the basis of previous published studies.

### 2.2 Inclusion and exclusion criteria

Included studies would be considered eligible if they met the population, intervention, comparator, outcomes, and study design criteria as follows:

Population: neonates with HIE;
Intervention: group with erythropoietin;
Comparator: group without erythropoietin;
Outcomes: The primary outcome was mortality rate. Secondary outcomes included neurodevelopmental disorders, brain injury, and cognitive impairment.
Study design: RCTs.

Exclusion criteria included observational studies, review articles, studies with a sample size <50, and studies with insufficient outcome data.

### 2.3 Data extraction

Data were extracted by review of each study for population, mean age, sex, follow-up duration, study design, publishing date, characteristics, and outcomes assessment. The 2 reviewers created a study-specific speadsheet in Excel for data collection. Data extraction was performed independently, and any conflict was resolved before final analysis. Any disagreements between the 2 reviewers were discussed and, if necessary, the third author was referred to for arbitration. If the data were missing or could not be extracted directly, authors were contacted by email. If necessary, we would abandon the extraction of incomplete data.

### 2.4 Risk of bias assessment

The Cochrane risk of bias tool was independently used to evaluate the risk of bias of included RCTs by 2 reviewers. The quality of RCTs was assessed by using following 7 items: random sequence generation, allocation concealment, blinding of participants and personnel, blinding of outcome assessment, incomplete outcome data, selective reporting, and other bias. Kappa values were used to measure the degree of agreement between the 2 reviewers and were rated as follows: fair, 0.40 to 0.59; good, 0.60 to 0.74; and excellent, ≥0.75.

### 2.5 Data synthesis

The present study was performed by Review Manager Software (RevMan Version 5.3, The Cochrane Collaboration, Copenhagen, Denmark). We used the Mantel-Haenzel method to calculate the pooled odds ratio. Odds ratio with a 95% confidence interval was assessed for dichotomous outcomes. *P* < .05 was set as the significance level. The heterogeneity was assessed by using the *Q* test and *I*^2^ statistic. When *I*^2^ ≥ 40%, it was considered to represent significant heterogeneity. All outcomes were pooled on random-effect model. The Z test was used to assess the overall effect.

## 3 Discussion

Multiple clinical trials have demonstrated the safety and efficacy of erythropoietin in improving neurodevelopmental outcomes in infants with HIE.^[8–10]^ A recent review involving randomized and non-randomized trials assessed neurodevelopmental disorders in infants with HIE. The authors did not explicitly define neurodevelopmental disorders, nor did they assess the risk and level of evidence for bias.^[11]^ Therefore, it is undoubtedly urgent to include only RCTs for more standardized systematic reviews and meta-analyses. The purpose of this study is to examine whether erythropoietin reduces the risk of death and improve neurodevelopmental disorders in infants with HIE. We hypothesized that group with erythropoietin would provide better therapeutic benefits compared with control group.

## Author contributions

**Conceptualization:** Yuan Zhao.

**Data curation:** Guang Yang, Zhimin Xue.

**Formal analysis:** Guang Yang, Zhimin Xue.

**Investigation:** Guang Yang, Zhimin Xue.

**Methodology:** Yuan Zhao.

**Project administration:** Yuan Zhao.

**Resources:** Guang Yang.**Software:** Zhimin Xue.

**Supervision:** Yuan Zhao.

**Validation:** Guang Yang, Zhimin Xue.

**Visualization:** Guang Yang.

**Writing – original draft:** Guang Yang.

**Writing – review & editing:** Yuan Zhao.
